# Comparative Analysis of Microbial Load in Smoked and Fried Tilapia (Chambo) From Wholesale and Retail Markets

**DOI:** 10.1002/mbo3.70094

**Published:** 2025-10-24

**Authors:** Patrick Ndovie, Agnes Banda, Noel Kapito, Sydney Namaumbo, Estone Malinda, Williams Mwatoma, Lecollins Mthilakuwiri, Macdonald Chabwera

**Affiliations:** ^1^ Department of Agriculture and Food Systems, Natural Resources College Lilongwe University of Agriculture and Natural Resources Lilongwe Malawi; ^2^ Department of Land and Water Resource, Natural Resources College Lilongwe University of Agriculture and Natural Resources Lilongwe Malawi; ^3^ Department of Animal Health and Livestock Development Central Veterinary Laboratory (CVL) Lilongwe Malawi; ^4^ Department of Food Science and Technology Bunda College, Lilongwe University of Agriculture and Natural Resources Lilongwe Lilongwe Malawi

**Keywords:** *Escherichia coli*, microbial contamination, *Oreochromis shiranus*, preservation methods, retailers, wholesalers

## Abstract

Fish, including *Oreochromis shiranus* (Chambo), is a vital protein source in Malawi, but consumption has declined. Due to its perishability, preservation methods like smoking and frying are common. This study compares the microbial load in smoked and fried tilapia from wholesale and retail markets. This cross‐sectional study compared the microbial load in smoked and fried tilapia (Chambo) from wholesale (Salima) and retail (Lilongwe) markets. Twenty fish samples (10 smoked and 10 fried) were collected, transported in sterile conditions, and analyzed for Total Aerobic Plate Count and coliforms using ISO‐standard methods. Data was log‐transformed and analyzed using two‐way analysis of variance, with significance set at *p* < 0.05. Fried tilapia from wholesalers had low levels of contamination, with most samples showing too few colonies to count, while some smoked samples displayed higher bacterial counts (33.75 ± 3.77 CFU/mL). Retailer samples showed a wider range of bacterial species, including *Escherichia coli* and *Pseudomonas putida*, suggesting potential hygiene concerns. Overall, bacterial loads were significantly higher in tilapia from retail markets compared with those from wholesalers (*p* < 0.05). The analysis showed higher microbial contamination in tilapia from retailers, with greater bacterial diversity, including *E. coli*, indicating poor hygiene. Wholesaler samples had minimal contamination. Although preservation methods showed no significant difference in microbial loads, retailer fish had significantly higher bacterial loads.

## Graphical abstract

This study investigates microbial contamination in fish samples from wholesalers and retailers in Salima and Lilongwe, Malawi. Significant microbial growth, observed in retailers rather than wholesalers, highlights potential health risks associated with fish handling practices. Findings emphasize the need for improved food safety protocols to mitigate contamination and ensure public health.

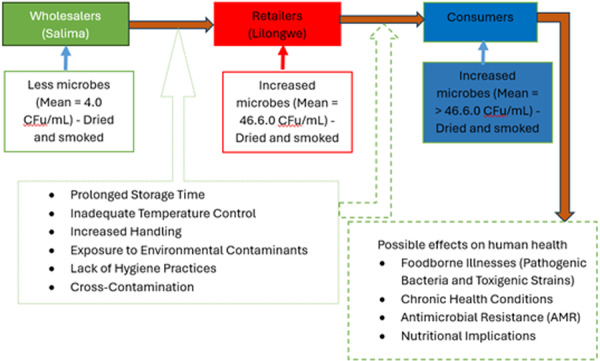

## 1 Introduction

Fish is a highly nutritious and affordable source of protein that plays a vital role in food security and nutrition, particularly in developing countries (Maulu et al. 2021, 2024; Obiero et al. 2019). *Oreochromis shiranus*, commonly referred to as *Chambo* in Malawi, is one of the most widely consumed freshwater fish due to its availability, affordability, and palatability (Chikowi et al. 2020). However, fish is highly perishable due to its high moisture content and nutrient composition, which create a favorable environment for microbial growth (Sheng and Wang 2021). Proper preservation methods, such as smoking and frying, are therefore essential to extend shelf life and reduce postharvest losses while ensuring food safety for consumers (Amit et al. 2017).

Smoking and frying are traditional preservation techniques used across many regions to reduce microbial load, enhance flavor, and improve the shelf stability of fish products (Akonor et al. 2016; Ryder et al. 2014). Smoking involves exposing fish to heat and smoke from burning wood, which dehydrates the product and inhibits microbial activity (El‐Sherif et al. 2021). On the other hand, frying fish, especially with oil smearing, uses high temperatures to reduce moisture content and kill pathogenic microorganisms (Teshome et al. 2022). Despite their widespread use, the effectiveness of these methods in reducing microbial load can vary depending on factors such as processing hygiene, storage conditions, and the handling practices at the wholesale and retail levels (Wogu et al. 2018).

The microbial load in fish products is a critical indicator of food safety, as contamination can result in foodborne illnesses and pose significant public health risks (Ryder et al. 2014). Poor handling and unhygienic conditions at different stages of the supply chain often lead to microbial contamination, particularly in informal markets, which dominate fish distribution in many low‐ and middle‐income countries (Bedane et al. 2022). Wholesale and retail markets can introduce different levels of contamination due to differences in storage conditions, processing practices, and duration of fish exposure to the environment (Ouedraogo et al. 2021). Previous studies have examined the influence of methods and storage environments on microbial safety of fish (Al‐Kahtani et al. 2019; Hossain et al. 2021). Nonetheless, there is a clear knowledge gap regarding direct comparisons of microbial loads in smoked and fried tilapia obtained from both wholesale and retail markets. This study is the first study to investigate how preservation method (smoking vs. frying) interacts with market type (wholesale and retail) to influence microbial contamination in *O. shiranus* (Chambo). By addressing this gap, the findings generate new evidence to inform targeted food safety interventions and strengthen consumer protection in Malawi's fish supply chain. Therefore, this study aimed to compare the microbial load of smoked and fried *O. shiranus* (Chambo) obtained from wholesale and retail markets.

## 2 Materials and Methods

### 2.1 Study Design and Sampling

**Figure 1:**
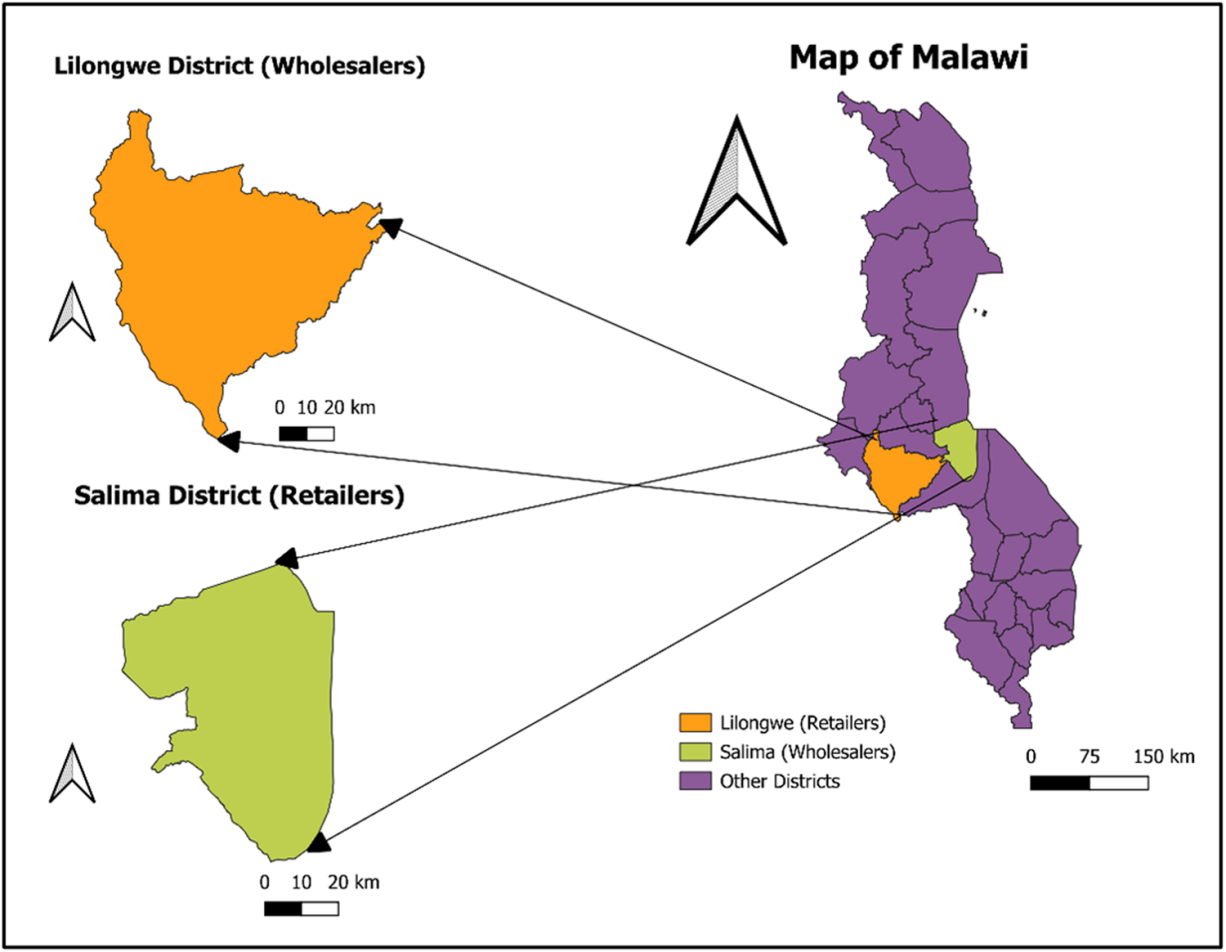
Research study areas: Salima and Lilongwe districts in Malawi. The map shows the locations of Salima and Lilongwe districts, where the study was conducted. Sampling sites for wholesalers and retailers are indicated with markers. Geographic data was developed by the corresponding author using QGIS.

**Figure 2:**
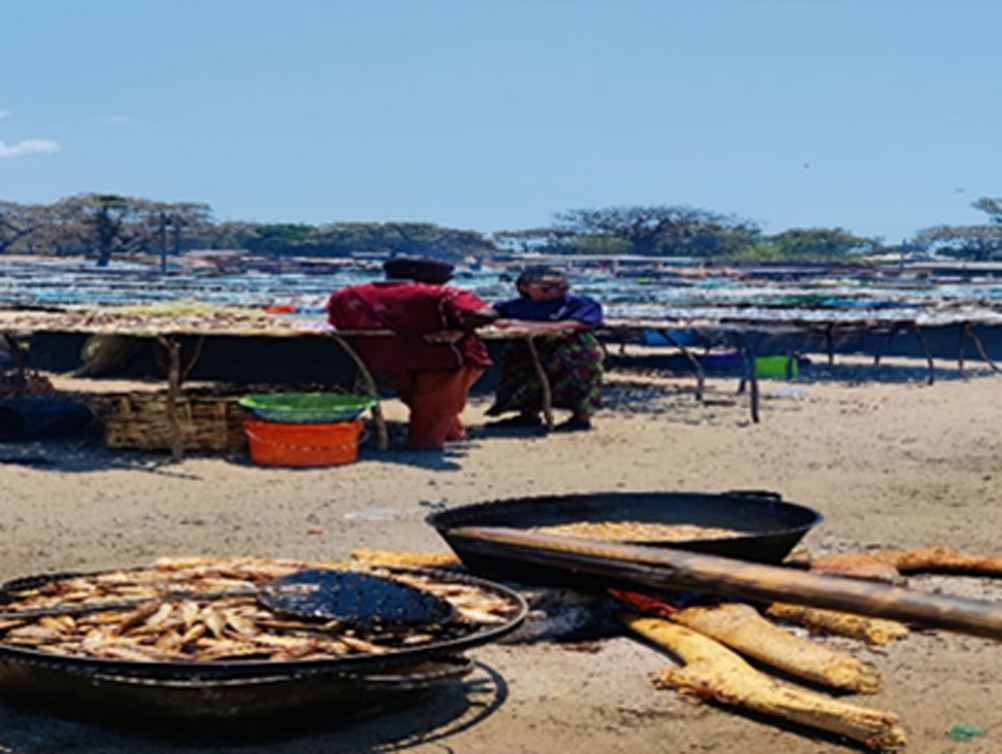
Sample collection from wholesalers in Salima. Photograph showing the process of collecting fish samples from wholesalers in the Salima district. Samples were handled aseptically and stored in sterile containers for transport to the laboratory.

**Figure 3:**
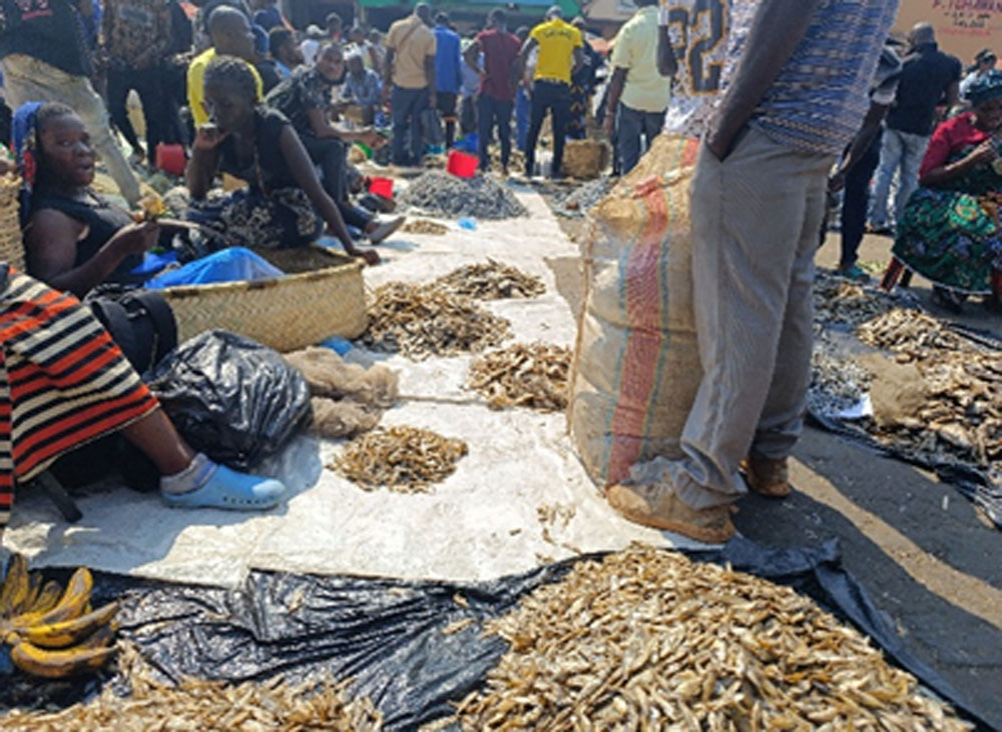
Sample collection from retailers in Lilongwe. Photograph illustrating the sample collection process at retail points in Lilongwe district. Samples were obtained in sterile conditions to prevent contamination and ensure integrity for microbiological analysis.

This study employed a cross‐sectional experimental design to compare the microbial load in smoked and fried *O. shiranus* (*Chambo*) obtained from wholesale and retail markets. Sampling was conducted over a period of two consecutive days from selected markets in Salima for wholesalers and Lilongwe for retailers (Figures 1, 2, 3). Before sampling, all fish outlets within the selected markets were listed, and samples were obtained the following day. The markets were purposively selected based on two criteria: (i) high volume of fish sales, which ensures consistent product availability, and (ii) accessibility, which facilitated timely sample collection. Within these markets, outlets were sampled using a convenience sampling approach, as this allowed practical access to smoked and fried tilapia being sold on the days of data collection. Larger markets were priotised as their higher fish turnover could impact microbial contamination levels due to factors, such as handling, storage conditions, and preservation methods.

### 2.2 Sample Collection

A total of 20 tilapia fish samples were collected, with equal representation of smoked and fried fish, as follows:

- 10 smoked tilapia samples: five from wholesale markets and five from retail markets.
- 10 fried tilapia samples: five from wholesale markets and five from retail markets.

The sample size of 20 was determined through a priori analysis conducted in SPSS version 26.0 (IBM Corp., Armonk, NY), assuming a medium effect size (Cohen's *d* = 0.5), a significance level of *α* = 0.05, and a desired statistical power of 80% (*β* = 0.20). These parameters were selected to ensure adequate sensitivity for detecting meaningful differences in microbial load between smoked and fried tilapia, while minimizing both Type I and Type II errors (Jacob 1988). Samples were collected by the principal investigator following a standardized procedure for fish sample collection. Both smoked and fried fish samples were collected in a wholesome manner before 5 a.m. to minimize postprocessing microbial growth, reduce environmental contamination, and ensure that the samples accurately reflected the microbial quality of fish as typically available to early‐morning customers.

Samples were collected under sterile conditions and immediately placed in well‐labeled, sterile zip‐lock specimen bags. To maintain the cold chain, the samples were transported to the laboratory within 24 h in a cool box containing ice packs, ensuring temperatures remained below 4°C to prevent microbial growth. A thermometer was placed inside the icebox to continuously monitor the temperature during transport. In the event of any temperature fluctuations, appropriate corrective measures, such as replenishing ice packs, were taken to maintain the cold chain. Sample transportation adhered to food microbiology safety guidelines (ISO 7218:2007).

### 2.3 Preparation of Samples

Upon arrival at the laboratory, each fish sample was handled with care and processed aseptically following the Central Veterinary Laboratory (CVL) Bacteriology Standard Operating Procedures (SOPs), which are based on internationally recognized protocols from the U.S. Food and Drug Administration's *Bacteriological Analytical Manual* (FDA 2023). A representative 25 g portion of fish tissue was gently excised using sterilized scalpels and forceps and homogenized in 225 mL of sterile buffered peptone water (BPW) using a stomacher (Stomacher 400, Seward, UK) for 2 min, producing a uniform suspension suitable for microbial analysis.

Serial dilutions of this homogenate were then prepared in sterile saline to allow precise counting of microbial populations. By following these standardized and carefully controlled procedures, all samples were handled consistently, contamination was minimized, and bacterial identification was both reliable and reproducible. This rigorous approach ensured that the microbial loads measured in smoked and fried *O. shiranus* accurately reflect the bacterial quality of the fish as available to consumers.

### 2.4 Microbial Analysis

The microbial load was determined through standard plate count methods following CVL Bacteriology SOPs and procedures outlined in ISO 4833‐1 (2013):

1. *Total Aerobic Plate Count:* Serial dilutions (10⁻¹–10⁻⁷) of the homogenized samples were prepared in sterile saline solution (0.85% NaCl). Aliquots (0.1 mL) of each dilution were spread onto a nutrient agar plate and incubated at 37°C for 24 h. Colonies were enumerated, and results were expressed as colony‐forming units per milliliter (CFU/mL).
2. *Coliform Count:* Diluted samples were plated onto Violet Red Bile Agar and incubated at 37°C for 24 h. Typical coliform colonies (pinkish red with precipitate zones) were counted.
3. Microbial (Bacterial) culturing incubation and identification

Bacterial isolation and identification were performed using both conventional and automated methods, with confirmatory testing carried out using Matrix‐Assisted Laser Desorption/Ionization Time‐of‐Flight (MALDI‐TOF) mass spectrometry. All procedures followed the CVL Bacteriology SOPs, which adopt internationally recognized protocols, including ISO 4833‐1 (2013). This approach ensured systematic, reliable, and accurate identification of bacteria associated with smoked and fried *O. shiranus*.

### 2.5 Inoculation, Culturing, and Incubation

**Figure 4:**
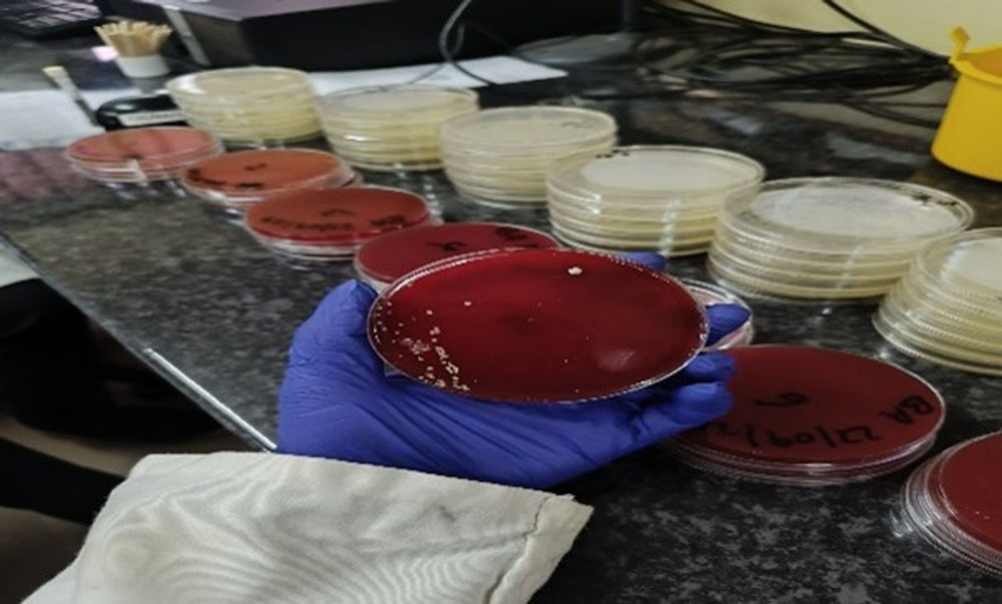
Microbial growth on blood agar from wholesalers' samples. Representative blood agar plate showing microbial growth from fish samples collected from wholesalers in Salima. Colonies exhibiting varying morphology indicate the presence of diverse bacterial species.

**Figure 5:**
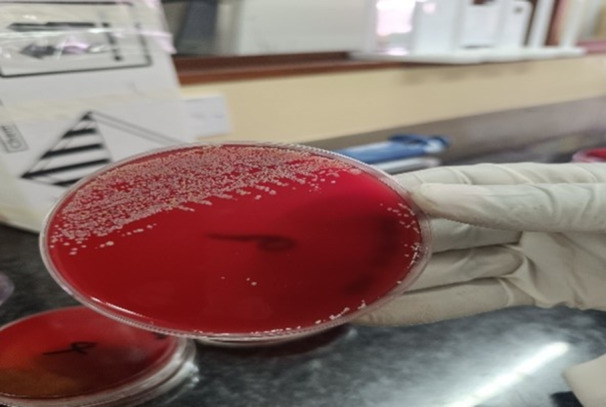
Microbial growth on blood agar from retailers' samples. Blood agar plate displaying bacterial growth from fish samples obtained from retailers in Lilongwe.

Sterile, ready‐prepared agar media, including Blood agar and MacConkey agar, were used for bacterial culture. The inoculation was done directly to the plate after dipping the loop into the zip‐lock bag containing the sample homogenate in peptone water. Using sterilized bacteriological loops, aliquots of the fish suspension were streaked onto the agar plates to isolate individual colonies. Care was taken to avoid cross‐contamination and to ensure proper separation of colonies. Inoculated plates were incubated at 37°C under aerobic conditions for 18–24 h, allowing visible colonies to develop. Representative examples of microbial growth observed on blood agar from wholesale and retail samples are shown in Figures 4 and 5, respectively.

### 2.6 Selection Criteria for Colony Identification

After bacterial isolation and purification, colonies were carefully selected for identification based on distinct and observable morphological characteristics, including size, shape, color, elevation, margin, and, when applicable, hemolysis patterns. Only colonies that appeared unique and clearly distinguishable were chosen for further analysis, ensuring that the diversity of bacterial populations in each sample was accurately represented.

1. *Microscopy:* Selected colonies were Gram‐stained and examined under a 100× objective lens to determine cell shape, arrangement, and Gram reaction, providing the first layer of classification.
2. *Biochemical Tests:* Purified colonies were subjected to a series of standard biochemical assays, including catalase, coagulase, oxidase, fermentation tests, motility, indole, urease, Triple Sugar Iron, Simmons citrate, and Sulfide Indole Motility tests, to assess their metabolic characteristics and support species‐level identification.
3. *Confirmatory Testing:* All isolates underwent MALDI‐TOF mass spectrometry, which provided precise, species‐level identification and cross‐validation against reference spectral libraries for enhanced accuracy.

This structured and rigorous approach ensured that only the most representative and distinct bacterial populations were analyzed, providing a reliable and reproducible picture of the microbial communities present in smoked and fried *O. shiranus*.

### 2.7 Quality Control Measures

To ensure the accuracy, reliability, and reproducibility of the results, a comprehensive set of quality control measures was implemented throughout the study:

- *Sterilization:* All media, reagents, and laboratory equipment were sterilized using High‐Temperature Short‐Time methods or autoclaving before use to eliminate potential contaminants.
- *Negative controls:* Sterile BPW and uninoculated media were included in each batch of samples to monitor for any inadvertent contamination during sample processing and analysis.
- *Duplicate testing:* Every assay, including plating, colony enumeration, and biochemical tests, was performed in duplicate to minimize technical errors and enhance result consistency.
- *Independent verification:* Colony morphology, counts, and biochemical test outcomes were independently assessed by two trained microbiologists, and any discrepancies were resolved by consensus to ensure accuracy.
- *Environmental monitoring:* Laboratory work surfaces, instruments, and tools were regularly cleaned and disinfected, and all procedures were conducted under strict aseptic conditions to prevent cross‐contamination.
- *Equipment calibration:* Incubators, thermometers, and the MALDI‐TOF system were regularly calibrated and maintained according to manufacturer specifications to ensure reliable operation.
- *Reference standards:* Biochemical tests and MALDI‐TOF identifications were validated using known reference strains and spectral libraries to confirm accuracy.

By implementing these rigorous and standardized measures, the study ensured that bacterial isolation and identification were trustworthy, reproducible, and reflective of the true microbial load in smoked and fried *O. shiranus*, providing results that accurately represent the microbial quality consumers would encounter.

### 2.8 Data Analysis

Microbial counts (CFU/mL) were log‐transformed for statistical analysis. Means and standard deviations for microbial loads were calculated for each sample category (smoked vs. fried and wholesale vs. retail). A two‐way analysis of variance was performed to test for significant differences in microbial loads between preservation methods (smoked vs. fried) and market types (wholesale vs. retail). Post hoc tests (Tukey's HSD) were used to identify pairwise differences. Statistical significance was set at *p* < 0.05. All analyses were conducted using SPSS version 26.0 (IBM Corp., Armonk, NY).

### 2.9 Ethical Considerations

Market vendors provided verbal consent, and they signed consent forms indicating their acceptance for sample collection, and confidentiality regarding the market locations and vendors was maintained.

## 3 Results

The analysis of microbial load in smoked and fried tilapia showed considerable variation in contamination levels across preservation methods and market sources (Table 1 and Figures 4 and 5).

**Table 1:** Bacterial load (CFU/mL) in fried and smoked tilapia samples from wholesalers and retailers.

S/N	Sample ID	Source	Preservation method	Mean ± SD (CFU/mL)
1	SA‐LF‐01	Wholesalers	Fried	TFTC
2	SA‐LF‐02	Wholesalers	Fried	TFTC
3	SA‐LF‐03	Wholesalers	Fried	TFTC
4	SA‐LF‐04	Wholesalers	Fried	TFTC
5	SA‐LF‐05	Wholesalers	Fried	1.3 ± 0.03
6	SA‐LF‐06	Wholesalers	Smoked	TFTC
7	SA‐LF‐07	Wholesalers	Smoked	TFTC
8	SA‐LF‐08	Wholesalers	Smoked	TFTC
9	SA‐LF‐09	Wholesalers	Smoked	TFTC
10	SA‐LF‐10	Wholesalers	Smoked	33.75 ± 3.77
11	LL‐BS‐11	Retailers	Fried	2.66 ± 0.28
12	LL‐BS‐12	Retailers	Fried	0.01 ± 0.00
13	LL‐BS‐13	Retailers	Fried	TNTC
14	LL‐BS‐14	Retailers	Fried	0.34 ± 0.12
15	LL‐BS‐15	Retailers	Fried	1.64 ± 0.42
16	LL‐BS‐16	Retailers	Smoked	0.78 ± 0.15
17	LL‐BS‐17	Retailers	Smoked	0.11 ± 0.01
18	LL‐BS‐18	Retailers	Smoked	0.02 ± 0.01
19	LL‐BS‐19	Retailers	Smoked	0.01 ± 0.00
20	LL‐BS‐20	Retailers	Smoked	TNTC

Abbreviations: CFU = colony‐forming unit, TFTC = too few to count, TNTC = too numerous to count.

Fried tilapia samples from wholesalers generally exhibited minimal contamination, with most samples showing too few to count (TFTC) bacterial colonies. However, one sample (SA‐LF‐05) had a bacterial load of 1.3 ± 0.03 CFU/mL. Similarly, smoked tilapia from wholesalers predominantly displayed TFTC results, except for one sample (SA‐LF‐10), which recorded a significantly higher load of 33.75 ± 3.77 CFU/mL. In contrast, fried tilapia samples from retailers exhibited a broader range of bacterial counts, including cases where the microbial load was too numerous to count (TNTC), highlighting severe contamination. Mean bacterial loads in retail fried samples ranged from 0.01 ± 0.00 to 2.66 ± 0.28 CFU/mL. Smoked tilapia from retailers exhibited bacterial loads between 0.01 ± 0.00 and 0.78 ± 0.15 CFU/mL, with one sample (LL‐BS‐20) recorded as TNTC, indicating substantial contamination. Furthermore, samples from retail markets consistently exhibited higher bacterial loads compared with those from wholesalers, suggesting increased microbial contamination during retail handling, storage, and exposure to ambient conditions.

The analysis of bacterial isolates revealed distinct patterns of microbial diversity across preservation methods and market sources (Table 2). In fried tilapia from wholesalers, bacterial isolates included *Bacillus cereus, Bacillus megaterium, Macrococcus caseolyticus*, and *Staphylococcus haemolyticus*. However, fried tilapia from retailers showed a greater diversity of bacterial species, including *B. megaterium, Enterobacter cloacae, Escherichia coli, Proteus mirabilis, Pseudomonas putida, M. caseolyticus, Psychrobacter faecalis*, and *Staphylococcus sciuri*.

**Table 2:** Distribution of bacterial isolates in fried and smoked tilapia by wholesaler and retailer.

Bacteria isolates	Wholesaler		Retailer	
	Fried	Smoked	Fried	Smoked
*Bacillus cereus*	+	−	−	−
*Bacillus megaterium*	+	−	+	−
*Brevibacillus* spp.	−	+	−	−
*Enterobacter cloacae*	−	−	+	−
*Escherichia coli*	−	−	+	+
*Klebsiella pneumoniae*	−	+	−	−
*Kocuria rhizophila*	−	+	−	−
*Macrococcus caseolyticus*	+	+	+	+
*Proteus mirabilis*	−	−	+	−
*Pseudomonas putida*	−	−	+	−
*Psychrobacter faecalis*	−	+	+	+
*Staphylococcus haemolyticus*	+	−	−	−
*Staphylococcus sciuri*	−	−	+	+

*Note:* + = present, − = absent.

Bacterial isolates were assessed in duplicate, and any conflicting results between replicates were recorded as present (“+”) to ensure potential contamination was captured.

For smoked tilapia, wholesalers exhibited bacterial isolates, such as *Brevibacillus* spp., *Klebsiella pneumoniae, Kocuria rhizophila, M. caseolyticus*, and *P. faecalis*. Smoked tilapia from retailers, on the other hand, contained *E. coli*, *M. caseolyticus, P. faecalis*, and *S. sciuri*. The presence of *M. caseolyticus* and *P. faecalis* across both preservation methods and market sources suggests their resilience and adaptation to diverse preservation conditions.

The comparison of mean bacterial loads (CFU/mL) between the two preservation methods—fried and smoked—showed no statistically significant difference (*t* = −0.13, df = 38, *p* > 0.05). Fried tilapia had a mean bacterial load of 23.9 ± 70.5 CFU/mL (95% CI: −9.15 to 56.85), while smoked tilapia recorded a slightly higher mean bacterial load of 26.7 ± 70.3 CFU/mL (95% CI: −6.16 to 59.61) (Table 3). Despite these numerical differences, the preservation method did not significantly influence microbial load. In contrast, significant differences were observed in bacterial load based on market source (*t* = −2.01, df = 38, *p* < 0.05). Tilapia sourced from wholesalers had a lower mean bacterial load of 4.0 ± 10.2 CFU/mL (95% CI: −0.76 to 8.79) compared with tilapia from retailers, which exhibited a significantly higher mean bacterial load of 46.6 ± 94.1 CFU/mL (95% CI: 2.51 to 90.6).

**Table 3:** Comparison of mean bacterial load (CFU/mL) across preservation methods and sources.

Variable	Level	Mean ± SD	SE	95% CI	*p* value	*T* value
Preservation methods	Fried	23.9 ± 70.5	15.8	−9.15 to 56.85	0.415	−0.13
	Smoked	26.7 ± 70.3	15.7	−6.16 to 59.61		
Source	Wholesalers	4.0 ± 10.2	2.3	−0.76 to 8.79	0.032	−2.01
	Retailers	46.6 ± 94.1	21.0	2.51 to 90.6		

## 4 Discussion

The microbial analysis revealed significant differences in bacterial contamination based on preservation methods and market sources. Fried tilapia samples from wholesalers exhibited low bacterial contamination, with most samples showing TFTC. Smoked *O. shiranus* from wholesalers displayed similar trends, except for one sample with a notably higher bacterial load. In contrast, retail samples consistently exhibited higher contamination levels, including instances where the bacterial load was TNTC. These findings align with previous studies highlighting the role of postprocessing handling and storage conditions in microbial contamination (Azuike et al. 2024; Khadka et al. 2017). Increased microbial loads at retail outlets are consistent with reports by Anihouvi et al. (2019) and Assogba et al. (2021), which attributed higher contamination to poor handling practices, exposure to environmental contaminants, and inadequate storage infrastructure. Additionally, the elevated microbial loads in retail samples may result from prolonged exposure to ambient temperatures and increased handling during storage and sales, as highlighted by Bhatia et al. (2024) and Bintsis (2018).

Bacterial diversity also varied across preservation methods and sources. Fried *O. shiranus* from wholesalers predominantly harbored *Bacillus* species, *M. caseolyticus*, and *S. haemolyticus*. In contrast, retailer samples showed a broader range of bacteria, including *E. coli* and *P. putida*, suggesting possible fecal contamination and suboptimal hygienic handling. The presence of these pathogens in the retailer samples indicates potential fecal contamination and poor hygiene during retail handling, as supported by findings from Falowo et al. (2020), Sani et al. (2023), and Al‐Mazrouei et al. (2024). Similarly, the presence of *E. coli*, a fecal indicator, aligns with evidence from studies identifying unhygienic conditions in retail markets as significant contributors to contamination Falowo et al. (2020), Sani et al. (2023), and Al‐Mazrouei et al. (2024).

Smoked tilapia from wholesalers had relatively fewer bacterial isolates, with resilience observed in species like *P. faecalis* and *M. caseolyticus*, which were common across all sources and preservation methods. This resilience could reflect the adaptive capabilities of these bacteria to varied storage conditions, as noted by Njagi et al. (2020) and Luo et al. (2023). These findings emphasize the critical need for better sanitation and the implementation of cold chain systems to minimize bacterial growth during storage and handling at retail outlets Odeyemi et al. 2019).

A total of 40 *O. shiranus* samples (20 fried and 20 smoked) were collected using a purposive sampling method from wholesalers and retailers in the study area. The comparison of mean bacterial loads between preservation methods did not reveal statistically significant differences, with both fried and smoked fish showing similar microbial contamination levels (*p* > 0.05). These findings align with Prabhakar et al. (2020), who reported that different preservation methods can be equally effective under optimal conditions. However, significant differences were observed between market sources, with retailer samples exhibiting higher bacterial loads than wholesaler samples (*p* < 0.05). This elevated contamination in retail fish is likely due to longer storage durations, inadequate temperature control, and frequent handling, consistent with observations by Pal et al. (2016) and Moyo et al. (2020). These results highlight the need for stricter hygiene practices at retail points, including regular handwashing by handlers, sanitization of surfaces and equipment, use of clean utensils, and proper personal protective equipment. Additionally, implementing improved packaging, maintaining temperature‐controlled storage, and reducing handling frequency can further minimize microbial contamination and ensure food safety (Kazi et al. 2022; Sperber and Doyle 2016).

This study highlights microbial contamination in fried and smoked *O. shiranus*, showing that preservation methods and market sources influence bacterial loads. Purposive sampling enabled the selection of relevant samples and highlighted the role of proper handling and storage in reducing contamination and ensuring food safety. Limitations include potential selection bias from purposive sampling and the focus on bacterial contamination only; future studies should consider random sampling, assess viral and fungal pathogens, and examine antibiotic resistance profiles, given the rising concern over antimicrobial resistance (Muddassir 2025). Expanding sample size and geographic coverage would further clarify regional variations and the effectiveness of preservation methods, informing strategies to improve fish product safety.

## 5 Conclusion

The study highlighted significant differences in microbial contamination levels between fried and smoked tilapia samples from wholesalers and retailers. While tilapia from wholesalers showed minimal bacterial contamination, retail samples exhibited higher contamination, including cases of TNTC bacterial loads, indicating poor handling and storage practices. Bacterial diversity was also greater in retail samples, with the presence of pathogens, such as *E. coli* and *P. putida*, suggesting potential fecal contamination and inadequate hygiene. Although no significant differences were found between preservation methods, tilapia from retailers had significantly higher bacterial loads than those from wholesalers, emphasizing the critical need for better sanitation, temperature‐controlled storage, and improved handling practices, particularly at retail outlets, to reduce contamination risks and ensure food safety.

However, the study was limited by its relatively small sample size and the cross‐sectional design, which may not fully capture seasonal or regional variations in microbial contamination. Future research should include larger, longitudinal studies across different regions and seasons, assess additional preservation methods, and evaluate the effectiveness of targeted interventions such as vendor hygiene training, improved packaging, and cold chain management in reducing microbial contamination.

## Author Contributions

**Patrick Ndovie:** conceptualization, writing – review and editing, writing – original draft, formal analysis, data curation, validation, supervision. **Agnes Banda:** writing – review and editing, writing – original draft, investigation, formal analysis, data curation, conceptualization. **Noel Kapito:** writing – review and editing. **Sydney Namaumbo:** writing – review and editing. **Estone Malinda:** formal analysis, data curation, conceptualization. **Williams Mwantoma:** investigation, formal analysis, data curation. **Lecollins Mthilakuwiri:** investigation, formal analysis, data curation. **Macdonald Chabwera:** writing – review and editing.

## Ethics Statement

The authors have nothing to report.

## Conflicts of Interest

The authors declare no conflicts of interest.

## Data Availability

The data that support the findings of this study are available in the supporting material of this article. All data generated or analyzed during this study are included in this published article.
